# Cell membrane cholesterol affects serotonin transporter efflux due to altered transporter oligomerization

**DOI:** 10.1038/s41380-025-03201-y

**Published:** 2025-09-02

**Authors:** Deborah Rudin, Dino Luethi, Marco Niello, Jae-Won Yang, Isabella Burger, Walter Sandtner, Ruth Birner-Gruenberger, Gerhard J. Schütz, Harald H. Sitte

**Affiliations:** 1 Institute of Pharmacology, Center for Physiology and Pharmacology, Medical University of Vienna, Waehringer Strasse 13A, 1090 Vienna, Austria; 2 Institute of Applied Physics, TU Wien, Lehargasse 6, 1060 Vienna, Austria; 3 Genetics of Cognition Laboratory, Neuroscience Area, Istituto Italiano di Tecnologia, Genova, Italy; 4 Institute of Chemical Technologies and Analytics, TU Wien, Getreidemarkt 9, 1060 Vienna, Austria; 5 Diagnostic and Research Institute of Pathology, Medical University of Graz, Neue Stiftingtalstrasse 6, 8010 Graz, Austria; 6 Hourani Center for Applied Scientific Research, Al-Ahliyya Amman University, Amman, Jordan; 7 Center for Addiction Research and Science-AddRess, Medical University Vienna, Waehringer Strasse 13A, 1090 Vienna, Austria

**Keywords:** Neuroscience, Molecular biology

## Abstract

The human monoamine transporters (MATs) for serotonin (SERT), dopamine (DAT), and norepinephrine (NET) play a key role in neurotransmission by transporting neurotransmitters from the synaptic cleft back into the neuron. MATs are embedded in the cell membrane’s lipid bilayer, encompassing cholesterol, phospholipids, and sphingolipids as main components. Membrane cholesterol association has been shown for all MATs impacting transporter conformation, substrate affinity, transport velocity, and turnover rates. In the present study, we compared the regulatory impact of cholesterol on the uptake and efflux function, binding affinity, and transporter oligomerization across all three MATs. We observed that cholesterol depletion impairs transporter-mediated uptake in human transporter-transfected HEK293 cells and reduces the binding affinity of all MATs. Electrophysiological investigations in SERT-expressing cells revealed that cholesterol alterations affect the transition of the transporter from the outward to the inward-facing conformation in the presence of substrate. Upon cholesterol depletion, FRET imaging and single molecule microscopy studies indicated altered oligomerization behavior exclusively for SERT. Interestingly, reduction of membrane cholesterol selectively increased amphetamine-induced efflux via SERT, while efflux via DAT and NET was reduced. This effect was diminished in a mutant with reduced PIP_2_ binding capacity. Hence, the increased efflux at SERT due to cholesterol depletion appears to depend on the ability of PIP_2_ to bind to SERT. Thus, we hypothesize that the interaction profile between cholesterol and MATs may fine-tune the transporter functionality and influence MAT-dependent disorders.

## Introduction

Human monoamine transporters (MATs) comprise transporters for serotonin (SERT), dopamine (DAT), and norepinephrine (NET) [1]. SERT, DAT, and NET are 12-pass transmembrane proteins that belong to the neurotransmitter:sodium symporter (NSS) and solute carrier 6 (SLC6) family [2]. MATs play a key role in neurotransmission by transporting neurotransmitters from the synaptic cleft back into the neuron thereby limiting the extent and duration of neuronal signaling [1, 3]. In addition, the application of certain psychostimulants (e.g. amphetamines), leads to MAT-mediated efflux of the corresponding monoamines [4].

MATs are embedded in the lipid bilayer of the cell membrane, which includes cholesterol, phospholipids, and sphingolipids as main components [5]. These components have been observed to regulate and impact membrane curvature and thus the function of membrane proteins [6, 7], including transporters and receptors involved in signaling processes [8]. Due to its size and shape, cholesterol mainly accumulates transiently in lipid rafts in specific sphingomyelin-rich microdomains that have unique biophysical properties [9]. Membrane proteins often accumulate in lipid rafts, because these domains represent thicker parts of the cell membrane that are better suited for the accommodation of membrane proteins such as neurotransmitter transporters [5]. However, since cholesterol is also more evenly distributed within the plasma membrane [10], interactions between membrane proteins and cholesterol are likely independent of lipid raft association. Although cholesterol only accounts for around 2% of total body mass, the cholesterol content of the brain makes up around 23% of the total cholesterol content of the body [11]. Hence, cholesterol represents one of the most abundant components of the cellular membrane in the brain and plays a vital role in brain development, neuronal function, and synaptogenesis [12, 13].

Membrane cholesterol association has been shown for all MATs (drosophila DAT: [14]; NET: [15, 16]; SERT: [17]). These interactions impact the conformational cycling and cholesterol depletion induces a more inward-facing conformation, which affects the apparent substrate affinity, maximum transport velocity, and substrate turnover rates (DAT: [18–20]; SERT: [21, 22]). To the best of our knowledge, there is no information on the regulation of NET functionality by cholesterol. More recently, it was observed that a reduction in membrane cholesterol by methyl-β-cyclodextrin (MβCD) negatively impacted DAT-mediated efflux [23]. In addition, it has been proposed that MATs are associated with cholesterol-rich lipid rafts [8], with cholesterol affecting MAT trafficking to the membrane (DAT: [24–26]; NET: [16, 27]; SERT: [28]).

It was shown by biochemical and spectroscopic approaches that MATs form higher oligomeric structures in the cell membrane [29–35]. The oligomerization of MATs has been proposed to serve as a trafficking quality control mechanism [36] and to be a prerequisite for drug-induced transporter-mediated efflux of transporter substrates [37]. Studies investigating the influence of phosphatidylinositol 4,5-bisphosphate (PIP_2_) on transporter function suggest that PIP_2_ binds directly to MATs and regulates their efflux behavior [34, 38–40]; furthermore, it was shown that PIP_2_ binding also promotes oligomerization [33, 41]. In contrast, the dimerization process of DAT does not depend on PIP_2_ or cholesterol [31], while NET forms dimers in a PIP_2_-dependent manner [34]. Most recently, the first evidence of NET-forming oligomers, in the dependence on cholesterol and PIP_2_, came from a study that used cryo-electron microscopy [15].

It has been demonstrated that membrane cholesterol regulates the hydrolysis and thus the function of PIP_2_ [42, 43]. Hence, it is possible that cholesterol indirectly affects the oligomerization of SERT, but not DAT, via PIP_2_. Thus, the regulation of the oligomeric states of neurotransmitter transporters at the plasma membrane of cells, particularly neurons, by cholesterol is a plausible hypothesis worth testing.

Numerous studies in humans suggest that abnormal cholesterol levels may be associated with various psychiatric disorders [44, 45]. Moreover, several MATs have been linked to psychiatric disorders: (i) SERT is involved in anxiety, depression, and autism, (ii) DAT is associated with ADHD, schizophrenia, and addiction, and (iii) NET is involved in depression and anorexia nervosa [1, 46–50]. Therefore, a better understanding of the molecular mechanisms of MAT function and how these mechanisms may be affected by altered cholesterol levels could provide valuable insight into potential clinical treatment options for psychiatric disorders.

In the current work, we investigated the role of cholesterol in all three MATs: DAT, NET, and SERT. Our goal was to compare the regulatory impact of cholesterol on the uptake and efflux functions, inhibitor binding affinity, and transporter oligomerization. We observed that cholesterol depletion selectively increased amphetamine-induced efflux and affected the oligomerization of SERT. In contrast, efflux via DAT and NET was reduced without effect on oligomeric species.

## Material and methods

### Material

[^3^H]5-HT (43.1 µCI mmol^−1^), [^3^H]dopamine (51.4 µCI mmol^−1^), [^3^H]nisoxetine (46.0 µCI mmol^−1^), [^3^H]WIN35428 (CFT) (82.6 µCI mmol^−1^), and Ultima Gold^TM^ XR liquid scintillation cocktail were purchased from PerkinElmer (Boston, MA, USA). [^3^H]MPP^+^ (60 µCi mmol^−1^) was obtained from American Radiolabeled Chemicals (St. Louis, MO, USA). Cell culture dishes were purchased from Sarstedt (Nuembrecht, Germany). Methyl-β-cyclodextrin (MβCD) and water-soluble cholesterol (cholesterol chelated with MβCD, 51 mg cholesterol/g MβCD) were from Sigma-Aldrich (St. Louis, MO, USA). All other chemicals and cell culture supplies were from Sigma-Aldrich (St. Louis, MO, USA).

### Cell culture

Human embryonic kidney (HEK) 293 cells (CRL-1573, ATCC, Manassas, Virginia, U.S.) stably expressing hSERT, hDAT, or hNET were maintained in humidified atmosphere (37 °C, 5% CO_2_) in Dulbecco’s Modified Eagle Medium (DMEM), supplemented with 10% heat-inactivated fetal calf serum (FCS), streptomycin (100 µg × 100 mL^−1^) and penicillin (100 U × 100 mL^−1^). Geneticin (50 µg × mL^−1^) was used as selection antibiotic. HEK293 cells have recently been authenticated using highly polymorphic short tandem repeat (STR) loci. STR loci were amplified with the PowerPlex® 16 HS System (Promega). Fragment analysis was done on an ABI3730xl (Life Technologies), and the resulting data were analyzed with GeneMarker HID software (Softgenetics). The results were matched with the Cellosaurus database to the cell line HEK293-DR-GFP-RAD51B-9 (RRID:CVCL_XX01): parental HEK293 cells (96.7%), stable HEK293 cell lines expressing YhDAT (90%)), YhNET (100%), and YhSERT (96.7%).

### Mutagenesis and transfection of SERT to reduce PIP_2_ binding capacity

Mutations in SERT were introduced using the Quickchange Lightning Kit (Agilent) with the following primers: K352A: TGGCTTTTGCTAGCTACAACGCGTTCAACAACAACTGCTACC; K460A: GTTCCCACACGTCTGCGCAGCGCGCCGGGAGCGGTT. SERT-K352A/K460A was transiently transfected into HEK293 cells and grown in DMEM supplemented with 10% heat-inactivated FCS, streptomycin (100 µg × 100 mL^−1^), and penicillin (100 U × 100 mL^−1^) in a humidified atmosphere (37 °C, 5% CO_2_). Transfections were carried out using Lipofectamine Plus (Invitrogen); 5 µg of plasmid encoding SERT-K352A/K460A or wild-type SERT was used for transient transfection of cells in 10 cm culture dishes. Cells were assayed 48 h after transfection.

### Membrane cholesterol depletion and replenishment

Freshly prepared solutions of MβCD or Cholesterol were dissolved in Krebs-HEPES buffer (KHB; 25 mM HEPES, 120 mM NaCl, 5 mM KCl, 1.2 mM CaCl_2_, 1.2 mM MgSO_4_, and 5 mM D-glucose, pH 7.3) and added to HEK293 cells expressing the MATs. The net amount of cholesterol in the cholesterol solution was calculated from the manufacturer’s lot data. A solution containing 50 µg/mL cholesterol had an approximate concentration of 1 mM MβCD as chelating agent. After incubation with various concentrations of MβCD or Cholesterol for 30 min at 37 °C, the buffer was aspirated, and the cells were washed twice with KHB. The cholesterol content in cell lysates was analyzed using the Amplex Red Cholesterol Assay Kit (Invitrogen) according to the manufacturer’s protocol.

### Cytotoxicity

Potential cytotoxic effects of cholesterol depletion were assessed with the CellTiter 96® Non-Radioactive Cell Proliferation Assay (MTT) (Promega GmbH, Walldorf, Germany) according to the manufacturer’s protocol. The cells were incubated with 6 and 10 mM MβCD for 30 min at 37 °C. Subsequently, MTT dye solution was added, and the plate was incubated for 2 h at 37 °C. After addition of the solubilization solution and incubation for 1 h, absorbance was recorded at 570 nm using a BioTek Synergy H1 hybrid multi-mode reader (Agilent Technologies Austria, Vienna, Austria) with integrated Gen5 microplate reader and imaging software. The relative cell viability was quantified by comparing the absorbance values of treated wells and compared to vehicle control wells.

### Uptake experiments

Experiments were performed as previously described [51, 52]. One day before the experiment, cells expressing the respective transporter were seeded onto poly-D-lysine-coated 96 well plates at a density of 30,000 cells per well in a final volume of 200 µL. For uptake experiments, the cell culture medium was replaced with 300 µL KHB with or without MβCD or cholesterol for 30 min at 37 °C. Next, the buffer was aspirated, and the cells were washed twice with KHB to remove any MβCD or cholesterol from the buffer. Then, the buffer was replaced with 0.1 µM [^3^H]5-HT (SERT), 0.1 µM [^3^H]dopamine (DAT), or 0.05 µM [^3^H]MPP^+^ (NET), and various concentrations of unlabeled substrate in a total volume of 50 µL. Uptake was terminated after 60 s by washing the cells with 200 µL ice-cold KHB. Subsequently, the cells were lysed with 200 µL Ultima Gold^TM^ XR liquid scintillation cocktail, and the amount of tritium in the cells was measured with a Wallac 1450 MicroBeta® TriLux liquid scintillation counter. Non-specific uptake was determined in the presence of 30 μM paroxetine (SERT), 30 μM cocaine (DAT), or 30 μM nisoxetine (NET). Monoamine uptake data were fitted by nonlinear regression, V_max_ and K_m_ values were calculated from Michaelis-Menten’s least-squares fit with GraphPad Prism (Prism 9.0.2, GraphPad Software, San Diego, CA, USA).

### Release experiments

Release experiments were conducted as described earlier [53]. In brief, cells expressing the respective transporter were seeded onto poly-D-lysine coated 96 well plates at a density of 30,000 cells per well in a final volume of 200 µL, 24 h before the experiment. The next day, the cells were preloaded with [^3^H]5-HT (hSERT), [^3^H]dopamine (hDAT), or [^3^H]MPP^+^ (hNET) by incubation with 0.05 µM of the respective tritiated neurotransmitter in KHB for 20 min at 37 °C. Then, the cells were washed twice with KHB supplemented with or without MβCD or cholesterol and incubated for 30 min at 37 °C. Next, the buffer was aspirated, and the cells were washed twice with KHB to remove any MβCD or cholesterol from the buffer. Subsequently, the cells were incubated with various concentrations of *d*-methamphetamine (METH) or *para*-chloroamphetamine (*p*CA) for 10 min at room temperature. Next, the supernatant was transferred to another 96-well plate and 200 µL Ultima Gold^TM^ XR liquid scintillation cocktail was added to the cells and supernatant. The amount of tritium in the cells and the supernatant was assessed with a Wallac 1450 MicroBeta® TriLux liquid scintillation counter. The release of tritiated neurotransmitters was expressed as a percentage of the total radioactivity of cells and supernatant together normalized to the basal efflux of untreated cells. Non-specific release was determined in the presence of 30 µM paroxetine, 10 µM mazindol, or 30 µM nisoxetine for SERT, DAT, or NET, respectively.

### Binding experiments

Membrane binding experiments were conducted as previously shown [51, 54]. Membranes for binding experiments were prepared from HEK293 cells stably expressing the respective transporter. The cells were rinsed twice with phosphate-buffered saline (137 mM NaCl, 2.7 mM KCl, 4.3 mM Na_2_HPO_4_, 1.5 mM KH_2_PO_4_, pH 7.4), harvested, and centrifuged at 400 g for 10 min at 4 °C. The pellets were resuspended in hypotonic HME buffer (20 mM HEPES NaOH pH 7.5, 1 mM EDTA, 2 mM MgCl_2_) and subsequently freeze-thawed twice using liquid nitrogen, followed by sonication for 10 s. Membranes were collected by centrifugation at 40,000 g for 15 min at 4 °C and resuspended in HME buffer. The membranes were pretreated with 6 mM MβCD, 0.1 mg/mL Chol, or vehicle for 30 min at 37 °C in binding buffer (20 mM Tris-HCl pH 7.5, 1 mM EDTA, 2 mM MgCl_2_, 120 mM NaCl, and 3 mM KCl). Next, the membranes were incubated with [^3^H]imipramine for SERT or [^3^H]CFT for DAT and NET in reactions of 0.3 mL. Nonspecific binding for SERT, DAT, and NET was determined in the presence of 10 µM paroxetine, 10 µM mazindol, and 30 µM nisoxetine, respectively. After 60 (SERT, DAT) or 30 min (NET), the reactions were terminated by rapid washing with ice-cold buffer and the membranes were collected onto glass fiber filters (Whatman GF/B). The samples were then dissolved in liquid scintillation cocktail and the amount of tritium bound to the membranes was determined by liquid scintillation counting.

### Whole-cell patch-clamp

HEK293 cells stably expressing hSERT were seeded into poly-D-lysine coated 29 mm dishes (Nunclon™, Thermo Scientific, Denmark) at low density 24 h before the measurement. Substrate-induced SERT-mediated currents were determined as previously described [55, 56]. In brief, cells were voltage-clamped using the whole-cell patch-clamp technique. For recordings of steady-state currents and peak current recovery, glass pipettes were filled with internal solution (133 mM potassium gluconate, 5.9 mM NaCl, 1 mM CaCl_2_, 0.7 mM MgCl_2_, 10 mM HEPES, 10 mM EGTA, adjusted to pH 7.2 with KOH, final potassium concentration 163 mM). For recordings of peak currents relaxation rates, glass pipettes were filled with potassium and sodium-free internal solution (NaCl and potassium gluconate were replaced by NMDG chloride adjusted to pH 7.2 with NMDG). During measurements, the cells were continuously perfused with external solution (140 mM NaCl, 3 mM KCl, 2.5 mM CaCl_2_, 2 mM MgCl_2_, 10 mM HEPES, 20 mM glucose, adjusted to pH 7.3 with NaOH). Currents were recorded using an Axopatch 700B amplifier and pClamp 11.2 software (MDS Analytical Technologies) at room temperature. For recordings of steady-state currents, cells were voltage-clamped to a holding potential of −70 mV and 5-HT was applied for 5 s with intermittent washing steps of 5 s. For recordings of peak currents relaxation rates, cells were voltage-clamped to a holding potential of 0 mV and 5-HT was applied for 5 s with intermittent washing steps of 5 s. For peak current recovery recordings, cells were voltage-clamped to a holding potential of −70 mV and 10 µM 5-HT was applied for 500 ms followed by increasing washout intervals and subsequent 5-HT test pulses. The resulting current amplitudes in response to the 5-HT application were quantified using Clampfit 10.6 software. For analysis, traces were filtered using a 100-Hz digital Gaussian low-pass filter.

### Confocal microscopy

HEK293 cells stably expressing YFP-hSERT, YFP-hDAT, or YFP-hNET were seeded into poly-D-lysine coated 29 mm dishes with 20 mm glass-bottom well (Cellvis, Sunnyvale, CA, USA) at a density of 20,000 cells per dish. The following day, the cell membranes were stained by incubation with 0.4% trypan blue for 1 min as previously described [34, 57–59]. Then, the dye was washed off and KHB supplemented with 10 mM MβCD or 0.1 mg/mL cholesterol was added. The dish was mounted above a 60× oil immersion objective on a Nikon A1R+ laser scanning confocal microscope system. Images were acquired using a 12 kHz resonant scanner. YFP was excited by a 488 nm laser line, while trypan blue was excited by a 561 nm laser line; the emission filters 525/50 nm and 595/50 nm were used. The emitted light was collected with a high-sensitivity GaAsP detector. The cells were imaged before and 30 min after the addition of MβCD, Chol, or blank.

### FRET imaging

Experiments to assess the potential of membrane cholesterol alterations to induce conformational changes of the MATs were conducted using HEK293 cells transiently expressing a human SERT construct with either a fluorescence donor (CFP) or acceptor (YFP) attached to the N terminus. The cells were seeded into 29 mm dishes with 20 mm bottom (# 1.5 glass; Cellvis) at a density of 10^5^ cells per dish two days before imaging. Before imaging, the cells were treated with MβCD, Chol, or vehicle for 30 min at 37 °C. FRET was measured with an iMIC inverted microscope (TILL Photonics GmbH) equipped with a 60× (1.49 NA) oil objective (Olympus). Fluorescence was excited with a 100 W Xenon Lamp (Polychrome, Till Photonics GmbH). The excitation light was filtered through 436/20 nm (CFP) or 514/10 nm (YFP) excitation filters (Semrock) and directed to the sample by a 442/514 dual-line dichroic mirror (Semrock). The emitted fluorescence light was filtered through a 480/40 nm and 570/80 nm dual emission filter (Semrock) and directed to a beamsplitter unit (Dichrotom, Till Photonics). The emission light was separated spatially according to the fluorescence wavelength using a 515 nm dichroic mirror (Semrock) and the resultant channels (<515 nm and >515 nm) were projected side by side onto an EMCCD chip (iXon Ultra 897Andor). Live Acquisition software (version 2.5.0.21; TILL Photonics GmbH) was used for recording. For optimal noise ratio and dynamic range, the camera was operated in 16-bit mode with a readout speed of 1 MHz. According to the manufacturer’s recommendation, an EM gain of 16 was applied to overcome the noise floor. Two images were taken per set (donor and acceptor emission after donor excitation and acceptor emission after acceptor excitation). Per cell treatment condition, ten sets were recorded on each experimental day and the images were then analyzed using Offline Analysis software (version 2.5.0.2; TILL Photonics GmbH). Background fluorescence was subtracted from each image and one region of interest (part of the plasma membrane) per cell was selected in the CFP channel. The average intensity of each region of interest was used for calculations. HEK293 cells expressing a CFP or YFP signal only were used to determine spectral bleed-through (BT) for the donor (0.57) and acceptor (0.04). Normalized FRET (NFRET) was calculated as follows:

$${NFRET}=\frac{{I}_{{FRET}}-{{BT}}_{{Donor}}\times {I}_{{Donor}}-{{BT}}_{{Acceptor}}\times {I}_{{Acceptor}}}{\sqrt{{I}_{{Donor}}\times {I}_{{Acceptor}}}}$$

Maximum FRET was determined using a fused CFP-YFP construct [29].

### Single-molecule microscopy

The mobile fraction and subunit stoichiometry of mGFP-hSERT were assessed as described previously [30, 41]. Fluorophores were excited at room temperature with 488 nm light from a directly modulated diode laser (LBX-488, installed in L6Cc laser combiner; Oxxius, Lannion, France). Custom-written software in LabVIEW (National Instruments, Austin, TX, USA) was used to adjust illumination intensity and timing. The laser beam was focused onto the back-focal plane of a Plan-Apochromat objective (100×/1.46 NA; Zeiss, Jena, Germany) mounted on an inverted Zeiss Axiovert 200 microscope. Appropriate emission filter sets (FF01-538/685-25; Semrock, Rochester, NY, USA, and zt488/640rpc; Chroma, Bellows Falls, VT, USA) were used to filter emission light. As detector we used a back-illuminated liquid nitrogen-cooled charge-coupled device camera (LNCCD1300-PB, Roper Scientific, Planegg, Germany). The excitation and photobleaching area was restricted by a slit aperture (Owis, Staufen im Breisgau, Germany). Stroboscopic illumination with excitation times of 5 ms was used; samples were excited and bleached in total internal reflection fluorescence (TIRF) mode.

Fluorescence recovery after photobleaching (FRAP) experiments were performed to determine the mobile fraction of mGFP-hSERT. A small area of the plasma membrane (∼50 µm^2^) was photobleached in TIRF mode and the fluorescence signal was measured every 10 s within a total time interval of 400 s. The data were fitted by a one-phase association curve.

$$I(t)/{I}_{0}={mf}\times (1-{e}^{-{Kt}})$$

*I*_*0*_ represents the fluorescence signal before photobleaching, *I(t)* the fluorescence signal at time *t*, *mf* the mobile fraction, and *K* the recovery rate constant.

As individual single molecule signals cannot be distinguished as well-separated spots at high surface densities, a method referred to as “thinning out clusters while conserving stoichiometry of labeling” (TOCCSL) was applied to examine SERT oligomerization [30, 41, 60]. A prebleach image was recorded and used to assess the surface density of mGFP-hSERT. After 50 ms, an aperture-confined region of the bottom plasma membrane was photobleached for 2000 ms, using a laser intensity of ∼2 kW/cm^2^. The TOCCSL image was acquired after 15 s recovery time with a reduced excitation laser intensity of 0.4–0.6 kW/cm^2^ and used for obtaining the probability distribution of single spot brightness values B, *ρ*(B). To determine the brightness of single mGFP-hSERT molecules, *ρ*_1_(B), we repeatedly bleached cells for 200 ms with a laser intensity of ∼2 kW/cm^2^, resulting in only a few remaining fluorophores. Laser intensities were determined in epifluorescence configuration.

TOCCSL images were analyzed using an in-house algorithm implemented in MATLAB (Mathworks, Portola Valley, CA, USA). Pixel counts were converted to photon counts by offset subtraction and multiplying with the inverse gain; individual diffraction-limited fluorescent signals were fitted by a Gaussian function. The probability distribution of single spot brightness values, *ρ*(B), was used to determine the oligomeric state of mGFP–hSERT. Applying autoconvolution, the monomer brightness distribution *ρ*_1_(B) was used to calculate the expected distributions for dimers *ρ*_2_(B) and higher-order oligomers. The overall single spot brightness distribution *ρ*(B) was then fitted by a linear combination of *ρ*_1_(B), *ρ*_2_(B), and higher-order oligomers.

$$\rho \left({{{\rm{B}}}}\right)={\sum }_{n=1}^{{n}_{\max }}{\alpha }_{n}\times {\rho }_{n}(B),{{{\rm{with\; normalization}}}}{\sum }_{n=1}^{{n}_{\max }}{\alpha }_{n}=1$$

Fitting *ρ*(B) yielded the fractions α_*n*_ of the different oligomeric states of co-diffusing transporter molecules carrying an active mGFP molecule. To calculate the standard deviations of each analysis, a bootstrapping method was applied, in which 50% of the data were analyzed in 100 repetitions.

### Photoclick cholesterol labeling

Photoclick cholesterol (Avanti, Alabaster, AL, USA) is a cholesterol analog with a photo-reactivatable diazirine group and an alkyne group for conjugation to azide by click chemistry [61]. Live cell photoclick cholesterol labeling was performed as described [61]. Briefly, HEK293 cells stably expressing YFP-tagged human SERT were treated with 20 µM photoclick cholesterol for 30 min at 37 °C in the presence and absence of 100 µM excess cholesterol as a competitor. The cells were washed twice with cold PBS and then irradiated under 365 nm UV light for 5 min. Cells treated with 20 µM photoclick cholesterol but not UV-irradiated were used as a negative control. Cells were then harvested and solubilized in lysis buffer containing 1% Triton X-100, 20 mM Tris-HCl (pH 8.0), 150 mM NaCl, 1 mM EDTA, 1 mM sodium orthovanadate, 5 mM NaF, 5 mM sodium pyrophosphate, and a protease inhibitor mixture. YFP-hSERT proteins were immunoprecipitated from cell lysates using GFP nanobody-conjugated agarose beads (GFP-TRAP; Chromotek, Planegg-Martinsried, Germany). Proteins were then released from the beads at 95 °C for 10 min in reduction and alkylation buffer (100 mM Tris-HCl; pH = 8.5, 1% sodium dodecyl sulfate, 10 mM tris(2-carboxyethyl) phosphine, 40 mM 2-chloroacetamide). The click chemistry reaction with released proteins was performed using the Click-&-Go Click Chemistry Reaction Buffer Kit (Click Chemistry Tools, Scottsdale, AZ, USA) with Cy5-azide and copper (II) sulfate according to the instructions with minor modifications. Following the click reaction, proteins were separated on SDS-PAGE gels, and Cy5 fluorescence was detected using a ChemiDoc MP imaging system (Bio-Rad, Hercules, CA, USA). Proteins were then transferred to a nitrocellulose membrane (Thermo Scientific) and immunostained with a rabbit anti-GFP polyclonal antibody (Thermo Scientific) followed by an HRP-conjugated goat anti-rabbit secondary antibody (Thermo Scientific). Chemiluminescence detection was conducted with the ChemiDoc MP system (Bio-Rad) and image analysis was carried out with Image Lab software (Bio-Rad).

### Statistical analysis

Experimental data were analyzed using Microsoft Excel and Prism software (GraphPad 10.0.1, Software Inc., La Jolla, CA, USA). V_max_ and K_m_ values obtained from uptake experiments were analyzed with two-way ANOVA followed by Dunnett multiple comparisons test. B_max_ and K_d_ values obtained from binding experiments were compared to control using the unpaired t-test. *k*_0.5_ values obtained from electrophysiological recordings were analyzed with two-way ANOVA followed by Dunnett multiple comparisons test. Transporter oligomerization effects were analyzed using two-way ANOVA followed by Sidak’s multiple comparison test. *, **, and *** indicate *P* < 0.05, <0.01, and <0.001, respectively. Changes in transporter efflux were analyzed using two-way ANOVA followed by Sidak’s multiple comparison test, *, **, and *** indicate *P* < 0.05, *P* < 0.01, and *P* < 0.001, respectively. The statistical tests used are given in each figure legend. Values are displayed as the mean and standard deviation. Values of p ≤ 0.05 were considered significantly different.

## Results

### Cholesterol depletion impairs transporter-mediated substrate uptake into HEK293 cells

**Fig. 1:**
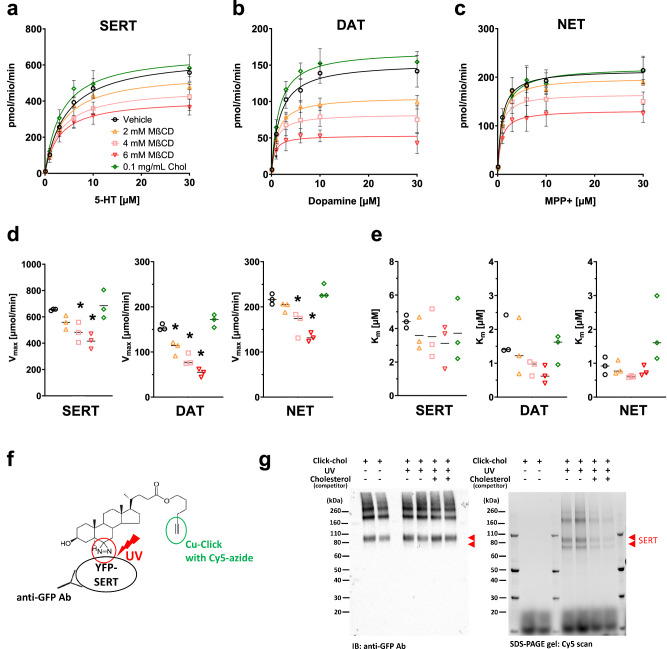
Effect of cholesterol alterations on MAT uptake. Transporter-mediated uptake in HEK293 cells stably expressing **a** SERT, **b** DAT, and **c** NET and thereof obtained **d** V_max_ and **e** K_m_ values. Cells were pretreated with vehicle control, MβCD, or Chol at the indicated concentrations for 30 min at 37 °C. Subsequently, the cells were incubated with tritiated substrate for 1 min in KHB. Non-specific uptake was determined in the presence of 30 µM paroxetine (SERT), 30 μM cocaine (DAT), or 30 μM nisoxetine (NET). Values represent mean ± SD of at least three experiments performed in triplicate. Data were analyzed with two-way ANOVA followed by Dunnett multiple comparisons test. * indicates *P* < 0.05 compared to the corresponding condition without MβCD or cholesterol. **f** Scheme o**f** UV light-induced cross-linking between click-cholesterol and YFP-SERT protein and subsequent conjugation to Cyanine5 (Cy5)-azide by copper (Cu)-catalyzed azide-alkyne cycloaddition (click) chemistry. **g** YhSERT cells were cultured with click-cholesterol (Click-chol) for 30 min in the presence (+) and absence (-) of normal cholesterol as a competitor, and then the cells were treated with (+) or without (-) UV irradiation. YFP-SERT proteins were immunoprecipitated with a GFP antibody and then conjugated with Cy5-azide. Immunoblot with anti-GFP antibody for immunoprecipitated YFP-SERT proteins (left panel) and Cy5 labeling of YFP-SERT with click-cholesterol (right panel) under the indicated conditions show specific click-cholesterol binding to SERT.

**Fig. 2:**
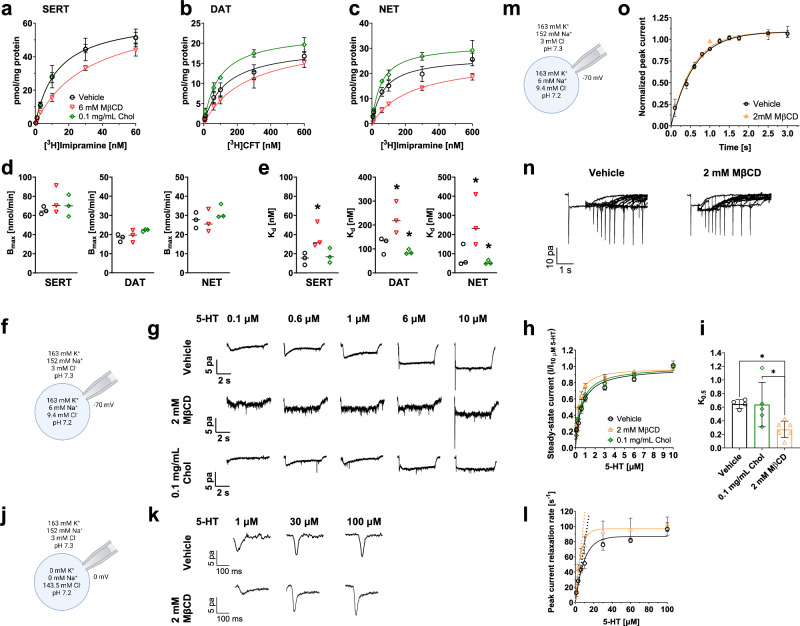
Membrane cholesterol content affects ligand binding to MATs and steady-state currents, peak current relaxation rate, and peak current recovery of SERT. Competition curves for **a** [^3^H]imipramine binding in HEK293 cells stably expressing SERT, **b** [^3^H]CFT binding in HEK293 cells stably expressing DAT, and **c** [^3^H]imipramine binding in HEK293 cells stably expressing NET. Cells were preincubated with 6 mM MβCD, 0.1 mg/mL Chol, or vehicle for 30 min at 37 °C. The curves were fitted by a linear regression one-site model. Data are shown as mean ± SD from at least three independent experiments. Nonspecific binding for SERT, DAT, and NET was determined in the presence of 10 µM paroxetine, 10 µM mazindol, and 30 µM nisoxetine, respectively. **d** B_max_ and **e** K_d_ values obtained from binding competition curves (a–c). **f** Delineative representation of the experimental conditions and **g** representative traces of steady-state currents generated by the application of the indicated 5-HT concentrations to HEK293 stably expressing GFP-tagged SERT in the presence of physiological ion gradients at a holding potential of −70 mV and pH 7.4. Cells were preincubated with 2 mM MβCD, 0.1 mg/mL cholesterol, or vehicle control for 30 min at 37 °C before measurement. **h** The amplitude of the steady-state current of SERT was determined at the indicated concentrations of 5-HT and normalized (as a ratio) to the current amplitude elicited by a pulse of 10 µM 5-HT in the same cell (n = 5 cells per substrate and condition). **i** *k*_0.5_ values calculated from the steady-state currents shown in (a). *P < 0.05 compared to control (unpaired t-test). **j** Delineative representation of the experimental conditions and **k** representative traces of 5-HT induced peak currents after application of 1, 30, and 100 µM at a holding potential of −0 mV. **l** The relaxation rate was determined at the indicated concentrations of 5-HT. Data points are shown as mean ± SD and were subjected to nonlinear least-squares curve-fitting to a hyperbola. The association rate constant *k*_on_ was calculated from the slope of the regression line fitted to the linear portion of the first three points (dashed lines). *k*_on_ and *k*_0.5_ rates are compiled in Table 3. **m** Delineative representation of the experimental conditions and **n** representative current traces were recorded at a holding potential of 0 mV. A pulse of 5-HT was applied to obtain a reference peak after which subsequent pulses of 5-HT were applied. Peak amplitudes after specific wash intervals represent the fraction of transporters available for 5-HT binding and thus recovery to the outward-open state of SERT. **o** The time course for 5-HT peak recovery was determined after pretreatment with 2 mM MβCD or vehicle control. Data represent mean ± SD and were fitted to the exponential for a mono-exponential rise to a maximum. Data were analyzed with two-way ANOVA followed by Dunnett multiple comparisons test. * indicates *P* < 0.05 compared to the corresponding condition without MβCD or cholesterol.

We performed uptake experiments using HEK293 cells stably expressing human SERT, DAT, and NET, respectively, to assess whether cholesterol depletion or replenishment of cell membranes affects their function. Cholesterol depletion by the application of MβCD significantly reduced the V_max_ of all tested MATs in a concentration-dependent manner (Table 1, Fig. 1a–e). Quantification of cholesterol depletion and replenishment showed that treatment with MβCD or cholesterol effectively altered the cholesterol content in MAT-expressing HEK293 cells (Suppl. Fig. S1a, b). After incubation for 30 min, the cholesterol concentration was significantly decreased by MβCD and increased by additional cholesterol in a concentration-dependent manner. Moreover, we showed that cholesterol interacts with SERT by UV light-induced cross-linking between click-cholesterol and YFP-SERT protein and subsequent conjugation to Cyanine5-azide by copper-catalyzed azide-alkyne cycloaddition chemistry (Fig. 1f, g). This is why cholesterol depletion can directly affect SERT function. Cell viability experiments confirmed that the reduced uptake after cholesterol depletion was not due to potential toxic effects (Suppl. Fig. S2). The observed decrease in V_max_ could result from either a decreased turnover rate or reduced surface expression of the transporters due to cholesterol depletion. Therefore, we performed confocal microscopy of HEK293-cells expressing YFP-tagged transporters and trypan-blue cell membrane staining to examine transporter surface expression levels. As shown in Suppl. Figs. S3–S6, neither cholesterol depletion nor replenishment affected the transporter surface expression of SERT (control 1.00 ± 0.13; depletion 1.00 ± 0.10; replenishment 0.95 ± 0.11), DAT (control 1.00 ± 0.37; depletion 0.97 ± 0.32; replenishment 0.97 ± 0.29), or NET (control 1.00 ± 0.31; depletion 1.07 ± 0.31; replenishment 1.00 ± 0.33). Based on these observations, we concluded that a decreased turnover rate ought to cause the observed decrease in V_max_. Indeed, the substrate turnover rates of SERT (calculated from the ratio of *V*_max_ of [^3^H]5-HT uptake (Fig. 1)/*B*_max_ of [^3^H]imipramine binding (Fig. 2)) [62] were 103 ± 2.3, 60 ± 2.1, and 105 ± 3.9 min^−1^ for control, 6 mM MβCD, and cholesterol replenishment, respectively. For DAT, the turnover rates were 79 ± 2.4 min^−1^ (control), 25 ± 1.3 min^−1^ (6 mM MβCD), and 75 ± 1.9 min^−1^ (cholesterol replenishment), and for NET 81 ± 2.6 min^−1^ (control), 51 ± 1.9 min^−1^ (6 mM MβCD), and 70 ± 2.3 min^−1^ (cholesterol replenishment).

**Table 1:** Effect of cholesterol depletion and replenishment in MAT expressing HEK293 cells.

	Vehicle	2 mM MβCD	4 mM MβCD	6 mM MβCD	0.1 mg/mL Chol
**SERT**					
V_max_ (μmol/min)	654 ± 14.8	557 ± 15.8	472 ± 18.3*	411 ± 14.5*	669 ± 25.1
K_m_ (μM)	4.4 ± 0.3	3.5 ± 0.3	3.2 ± 0.4	2.9 ± 0.4	3.4 ± 0.4
**DAT**					
V_max_ (μmol/min)	154 ± 4.6	106 ± 5.8*	82.6 ± 3.6*	53.2 ± 2.8*	170 ± 4.2
K_m_ (μM)	1.7 ± 0.2	1.1 ± 0.3	0.8 ± 0.2	0.6 ± 0.2	1.5 ± 0.2
**NET**					
V_max_ (μmol/min)	215 ± 7.0	199 ± 4.9	166 ± 5.7*	132 ± 5.0*	221 ± 7.1
K_m_ (μM)	0.9 ± 0.1	0.8 ± 0.1	0.6 ± 0.1	0.7 ± 0.2	1.1 ± 0.2

Cells were pretreated with vehicle control, MβCD, or cholesterol at the indicated concentrations for 30 min at 37 °C. Treated cells were subsequently incubated with tritiated substrate for 1 min (SERT, DAT, NET) in KHB. Values represent mean ± SD of at least three experiments performed in triplicate. *P < 0.05 compared with control (ANOVA, Dunnett multiple comparisons test).

### Cholesterol alterations affect the binding affinity of MATs

Based on previous studies that reported a change in conformation of neurotransmitter transporters due to cholesterol depletion [18–23, 63], we assessed the effect of cholesterol alterations on the binding affinity of SERT, DAT, and NET in membranes prepared from cell lines stably expressing these transporters. After cholesterol depletion, SERT bound [^3^H]imipramine with lower affinity than untreated SERT, whereas there was no difference between cholesterol replenishment and untreated SERT (Fig. 2a, Table 2). This finding is consistent with the notion that cholesterol depletion stabilizes the inward-facing conformation of SERT, which aligns with the results of previous studies [22, 64]. Similar to the results observed with SERT, DAT, and NET bound [^3^H]CFT and [^3^H]imipramine, respectively, with lower affinity than the corresponding untreated transporters (Fig. 2b, c, Table 2). However, cholesterol replenishment increased the affinity of DAT and NET for [^3^H]CFT and [^3^H]imipramine, respectively (Fig. 2b, c, Table 2). Hence, cholesterol depletion appears to stabilize the inward-facing conformation of SERT, DAT, and NET, while cholesterol replenishment stabilizes the outward-facing conformation of DAT and NET.

**Table 2:** Effect of cholesterol depletion and replenishment on the binding affinity of MATs.

	Vehicle	6 mM MβCD	0.1 mg/mL Chol
**SERT**			
K_d_ (µM)	13.3 ± 1.9	32.3 ± 2.8*	13.3 ± 1.2
**DAT**			
K_d_ (µM)	128 ± 18.8	263 ± 41.4*	91.1 ± 8.9*
**NET**			
K_d_ (µM)	70.1 ± 10.4	237 ± 20.9*	54.3 ± 5.4*

Cells were pretreated with vehicle control, MβCD, or cholesterol at the indicated concentrations for 30 min at 37 °C. Values represent mean ± SD of at least three experiments performed in duplicate. *P < 0.05 compared with vehicle control (ANOVA, Dunnett multiple comparisons test).

### Cholesterol alterations affect 5-HT-mediated currents

We used electrophysiological recordings of SERT to elucidate further the effect of cholesterol alterations on the SERT transport cycle. The substrate-induced current through SERT comprises two components: (i) an initial peak current, which reflects the transition from the substrate-loaded outward- to the substrate-loaded inward-facing conformation of the transporter, and (ii) the steady-state current that is produced by an uncoupled conducting state and reflects cycling of the transporter in the forward direction. As shown in Fig. 2f–i, cholesterol depletion with 2 mM MβCD resulted in a left shift of the dependence of the steady state current amplitude on the substrate concentration (Vehicle *k*_0.5_ = 0.64; 0.1 mg/mL Chol *k*_0.5_ = 0.29; 2 mM MβCD *k*_0.5_ = 0.57) (Table 3). Due to increased instability of the cellular membrane, higher concentrations of MβCD could not be assessed.

**Table 3:** Steady-state currents and peak current relaxation rates of SERT are affected by membrane cholesterol contents.

	*k*_0.5_	*k*_on_ [10^6^ M^−1^s^−1^]
Vehicle	0.64 ± 0.06	7.78 ± 1.23
2 mM MβCD	0.29 ± 0.12	11.65 ± 2.61
0.1 mg/mL Chol	0.57 ± 0.33	-

*k*_0.5_ and *k*_on_ values were obtained from steady-state currents and peak current relaxation rate measurements of SERT, respectively.

### Cholesterol replenishment did not affect the peak current of SERT

Next, we recorded the peak current of SERT to examine whether cholesterol alterations affect the isomerization from the outward to the inward-facing conformation in the presence of substrate. For this, we determined the peak current relaxation rate, which reports on the rates of two reactions [65, 66]: (i) the apparent substrate binding rate *k*_app_, which is rate-limiting at low substrate concentrations, and (ii) the isomerization step from the outward to the inward facing state which is rate-limiting at high substrate concentrations. We studied the peak current in isolation by using potassium- and sodium-free internal solution, since binding of intracellular potassium to the inward-facing conformation of SERT is required for the induction of the steady-state current [66]. The obtained values for the peak current relaxation were plotted as a function of the 5-HT concentration and fit to a mono-exponential function. The slope of this relation, at low substrate concentrations, allows for estimating the substrate association rate (k_on_). Cholesterol depletion with 2 mM MβCD increased the peak current relaxation rate and led to an almost doubled association rate constant (*k*_on_) (Fig. 2j–l and Table 3). However, recordings of the peak current recovery showed that cholesterol depletion did not affect the time course of peak current recovery (Fig. 2m–o). The increased *k*_on_ of the substrate after cholesterol depletion is in line with the previously observed decreasing K_m_ values during uptake experiments with increasing MβCD concentrations (Fig. 1a–e). Recordings of the peak current recovery with two pulse protocols showed that cholesterol depletion did not affect the time course of peak current recovery (Fig. 2m–o). The peak current recovery rate is a surrogate of the substrate turnover rate [66].

### Cholesterol alterations affect SERT oligomerization

**Fig. 3:**
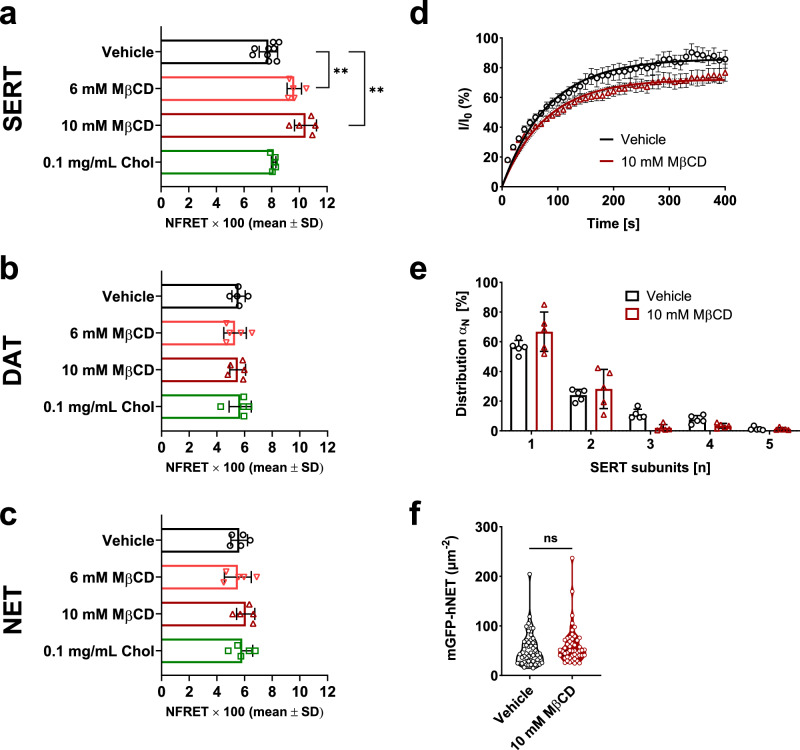
Effect of cholesterol alterations on monoamine transporter oligomerization and subunit stoichiometry of SERT. **a–c** HEK293 cells transiently transfected with human SERT, DAT, or NET with a fluorescence donor (CFP) or acceptor (YFP) attached to the N-terminus were used. Cells were pretreated with vehicle, 6 mM or 10 mM MβCD, or 0.1 mg/mL Chol. Average NFRET values (×100) are presented as mean ± SD; per cell treatment condition, ten sets were recorded on each experimental day. **d** Mobile fraction of SERT determined by FRAP microscopy. Data points represent averages of eleven cells (±SD), which were fitted by a one-phase association curve. **e** Subunit distribution of SERT at the plasma membrane. Each data point represents an independent experiment with TOCCSL runs on ten cells. Bars represent mean ± SD. **f** Surface density of SERT after cholesterol depletion was statistically indifferent to control cells. Data were analyzed with two-way ANOVA followed by Sidak’s multiple comparison test. *, **, and *** indicate *P* < 0.05, < 0.01, and < 0.001, respectively, when compared to vehicle.

Several transporters of the SLC6 family have been shown to form oligomeric complexes in living cells [29–31, 33–35, 67] and lipids may affect oligomer formation [41]. Hence, we assessed whether cholesterol alterations change the oligomerization of MATs. We recorded FRET in HEK293 cells transiently transfected with MATs with either a fluorescence donor (cyan fluorescent protein, CFP) or acceptor (yellow fluorescent protein, YFP) attached to the amino terminus. The formation of oligomers increases FRET compared to transporters in the monomeric state. As shown in Fig. 3a–c and Suppl. Fig. S7 cholesterol depletion led to a concentration-dependent increase in NFRET values in SERT, but not in DAT and NET. In contrast, cholesterol replenishment did not affect the recorded NFRET values in either transporter. Hence, only SERT seems to change its oligomerization behavior upon decreased membrane cholesterol.

Since the FRET microscopy experiments do not provide insights into the subunit stoichiometry of SERT after cholesterol depletion, we assessed the subunit stoichiometry of SERT on the single molecule level after depleting membrane cholesterol with 10 mM MβCD. As “thinning out clusters while conserving stoichiometry of labeling” (TOCCSL) experiments report only on the oligomerization of mobile molecules [60], we first determined the mobile fraction of SERT using fluorescence recovery after photobleaching (FRAP) experiments (Fig. 3d). FRAP experiments revealed a reduction of the mobile fraction from 86% (untreated) to 72% under cholesterol-depleted conditions. Next, we addressed the oligomeric state of SERT. Under control conditions, mobile SERT molecules formed quaternary arrangements up to tetramers (Fig. 3e). The transporter densities of the imaged cells were unaffected under both conditions (Fig. 3f). Taken together, our FRET data indicates that cholesterol depletion leads to a general increase in SERT oligomerization. Our TOCCSL and FRAP data show that these oligomers are largely immobilized. Additional mobile SERT monomers and dimers are still present after cholesterol depletion.

### Cholesterol alterations affect transporter-mediated efflux differently depending on the MAT

**Fig. 4:**
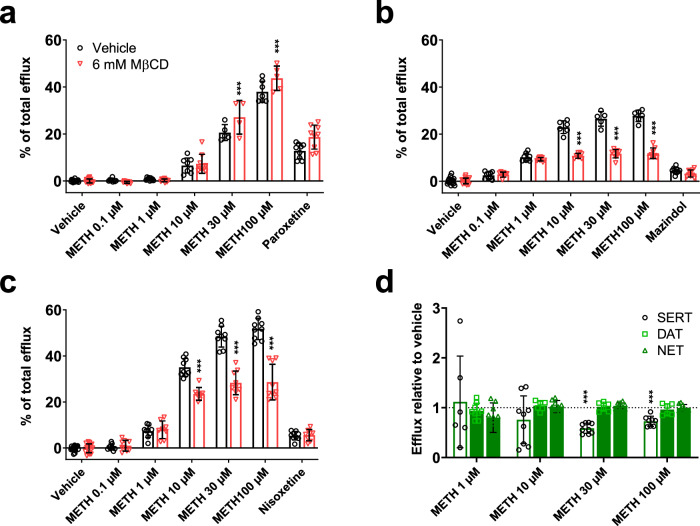
Effect of cholesterol alterations on MAT efflux. Substrate release in HEK293 cells stably expressing SERT **a**, DAT **b**, or NET **c** was induced by *d*-methamphetamine (METH) in the absence (black bars) or presence (red bars) of 6 mM MβCD after preloading the transporter-transfected cells with radiolabeled substrate. Substrate release was normalized to basal efflux. The transporter blockers paroxetine (30 µM) for SERT, mazindol (10 µM) for DAT, and nisoxetine (30 µM) for NET were used to assess non-specific efflux. **d** Substrate release in HEK293 cells stably expressing SERT, DAT, or NET was induced by *d*-methamphetamine (METH) in the absence or presence of 0.1 mg/mL cholesterol. Data are normalized relative to vehicle efflux. Data were analyzed with two-way ANOVA followed by Sidak’s multiple comparison test. *, **, and *** indicate *P* < 0.05, *P* < 0.01, and *P* < 0.001, respectively, when compared to the corresponding condition without MβCD or Chol. All data are presented as mean ± SD from at least three experiments performed in triplicate.

Oligomerization has been suggested as a prerequisite for transporter-mediated efflux in MATs [37]. Since we observed changes in oligomerization due to cholesterol depletion in SERT, but not in DAT and NET, we next investigated the effect of cholesterol alterations on the transporter-mediated efflux of substrate. Efflux was induced by METH in the same MAT-expressing cell lines used for uptake experiments. Interestingly, cholesterol depletion increased the substrate efflux induced by METH selectively at SERT, whereas it was decreased at DAT and NET (Fig. 4a–c). This observation represents a remarkable contrast to our previous results, where cholesterol depletion similarly affected the transporter uptake function and binding affinities in all three transporters. Furthermore, cholesterol replenishment decreased the substrate efflux at SERT while no effect was observed at DAT and NET (Fig. 4d). These findings point to a striking difference between SERT and the other two transporters regarding the role of cholesterol during substrate efflux.

We performed additional experiments to verify the increase in SERT efflux. First, we sought to exclude potential biasing effects of the transporter substrate 5-HT, which is why we used the non-selective substrate MPP^+^ in further experiments. At physiological pH, the natural substrate 5-HT is only partially charged, whereas MPP^+^ always carries a positive charge [68]. Hence, while uncharged 5-HT may diffuse across the cell membrane leading to pseudo-efflux, MPP^+^ needs to be actively transported during efflux, precluding efflux by membrane diffusion [69]. Changes in efflux due to different cellular radioactivity levels as a result of cholesterol depletion could be ruled out because the total radioactivity measured at the end of each experiment was similar in control and cholesterol-depleted cells (Suppl. Fig. S8). Similar to the results obtained with tritiated 5-HT, efflux induced by METH in [^3^H]MPP^+^ preloaded cells was increased after cholesterol depletion (Suppl. Fig. S9). Hence, a potential biasing effect of the substrate could be excluded. In summary, the altered oligomerization due to cholesterol depletion may indeed be responsible for the enhancement of efflux at SERT. Therefore, we moved on to examine the role of cholesterol in SERT oligomerization more closely.

### The oligomeric state of SERT affects the transporter-mediated efflux

**Fig. 5:**
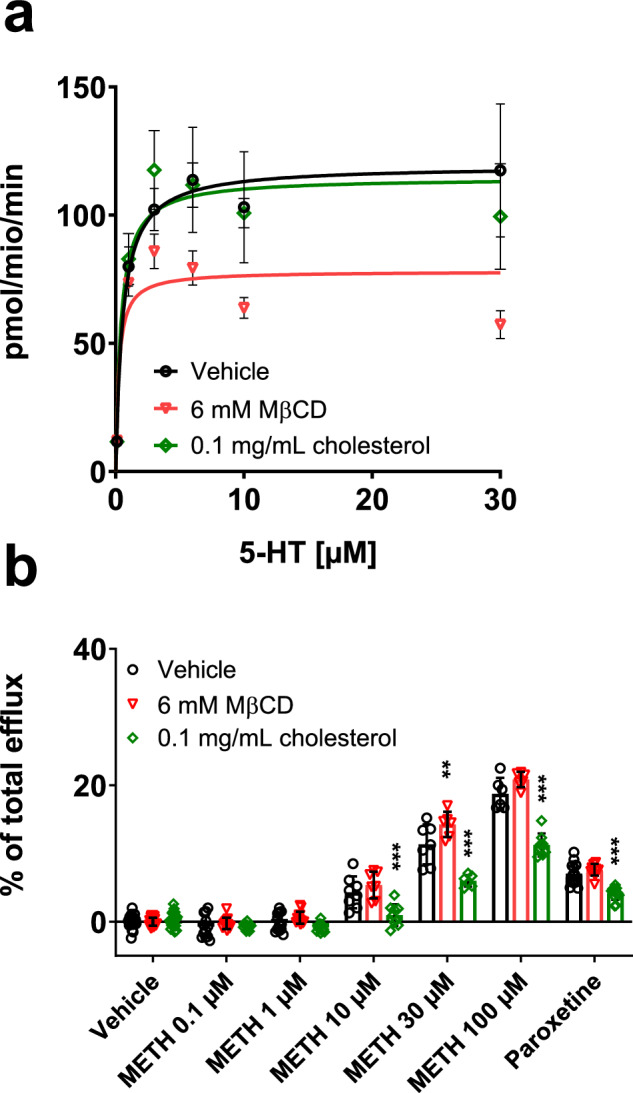
Effect of cholesterol alterations on monoamine transporter uptake and efflux of YhSERT_KKAA. **a** Transporter-mediated uptake in HEK293 cells expressing SERT_KKAA. Non-specific uptake was determined in the presence of 30 µM paroxetine. **b** Substrate release in HEK293 cells expressing SERT_KKAA was induced by *d*-methamphetamine (METH) in the absence (black bars) or presence (red bars) of 6 mM MβCD or in the presence of 0.1 mg/mL cholesterol (green bars) after preloading the transporter-transfected cells with radiolabeled serotonin. Data were analyzed using two-way ANOVA followed by Sidak’s multiple comparison test. *, **, and *** indicate *P* < 0.05, *P* < 0.01, and *P* < 0.001, respectively, when compared to the corresponding condition without MβCD or cholesterol. All data are presented as mean ± SD from at least three experiments performed in triplicate. Corresponding uptake and release experiments in HEK293 cells expressing wild-type SERT are shown in Figs. 1a and 4a, respectively.

Alterations in membrane lipids have been shown to impact transporter-mediated efflux. Buchmayer and colleagues demonstrated that the amino acid residues K352 and K460 are crucial for PIP_2_ binding to SERT [38]. Moreover, the mutation of both residues to alanine (SERT_KKAA) resulted in a substantial decrease in PIP_2_-induced effects on stimulant-induced 5-HT efflux, attributed to the reduced PIP_2_ binding capacity [38]. Additionally, single-molecule brightness analysis yielded an oligomeric distribution with a lower fraction of trimers and tetramers but more monomers and dimers compared to wild-type SERT [41]. Therefore, we used SERT_KKAA to elucidate whether potentially increased PIP_2_ levels due to cholesterol depletion are responsible for the increased stimulant-induced 5-HT efflux. As shown in Fig. 5a, SERT_KKAA uptake was not affected by the mutation of the two residues, and cholesterol depletion reduced the uptake capacity similarly as observed in wild-type SERT. However, the effect of cholesterol depletion on the METH-induced 5-HT efflux was negligible except for 30 µM METH treatment in SERT_KKAA (Fig. 5b). Interestingly, the decreased substrate efflux due to cholesterol replenishment was more pronounced in SERT_KKAA than in SERT (Fig. 5b). As shown in Suppl. Fig. S10, neither cholesterol depletion nor replenishment affected the transporter surface expression of SERT_KKAA. Hence, the increased efflux at SERT due to cholesterol depletion appears to rely on the ability of PIP_2_ to bind to SERT.

## Discussion

In the current study, we investigated the impact of cholesterol on the function of all three MATs. We examined how this lipid affects substrate uptake, amphetamine-induced substrate release, inhibitor binding, and transporter oligomerization. While cholesterol depletion uniformly decreased transporter V_max_ and IC_50_ values of orthosteric inhibitors of the MATs, we also identified interesting differences. Specifically, we found that reduced cholesterol levels augmented amphetamine-induced substrate release through SERT but inhibited substrate efflux mediated by DAT and NET. Another notable idiosyncrasy was that reducing the membrane cholesterol concentration caused higher-order SERT oligomers to form, while this effect was not observed for the other two MATs.

PIP_2_ and cholesterol are both lipids that affect amphetamine-induced substrate release. While PIP_2_ interacts with intracellular transporter segments, cholesterol binds to the hydrophobic core of the transporter [17]. In SERT, reduced cellular PIP_2_ and cholesterol levels have opposite effects on amphetamine-induced substrate release (i.e., a reduction of release upon PIP_2_ depletion and an enhancement of release upon cholesterol depletion). However, this is not the case for DAT and NET, where the reduction of either one of these lipids lowers substrate efflux [34, 38, 39].

It is conceivable that in SERT, PIP_2_ and cholesterol interact with each other allosterically, increasing their affinity for the transporter when bound together. If true, the effect of cholesterol on substrate release could be due to its role in stabilizing the PIP_2_-SERT interaction. To test this hypothesis, we used the mutant SERT_KKAA, which has a reduced PIP_2_ binding capacity and a reduced response to amphetamines [38]. In contrast to our observations in wild-type SERT, cholesterol depletion did not augment the amphetamine-induced release in SERT_KKAA expressing cells, consistent with the idea that the cholesterol-dependent alterations of substrate release are due to a modulatory effect of this lipid on the PIP_2_-SERT interaction. Previous studies showed that pharmacologically reduced PIP_2_ levels due to phospholipase C activation or phosphatidylinositol 4-kinase inhibition reduced stimulant-induced SERT release [41]. Hence, modulation of PIP_2_ levels could rescue the effects of decreased cholesterol levels. For NET, it has been most recently shown that it exists in oligomeric form, dependent on cholesterol and PIP_2_ [15] – while the same has not been elucidated for DAT or SERT [70, 71].

In addition to the lipid composition of the cell membrane, the oligomeric state of the MATs has also been shown to impact amphetamine-induced substrate release [33, 36, 37]. We observed increased intermolecular FRET for SERT at reduced cholesterol levels (utilizing NFRET measurements employing CFP- and YFP-labeled SERT wild-type constructs). These changes in observed FRET may be attributed to alterations in the oligomeric state of the transporters or changes in the mutual orientation of the fluorescent proteins. Our analysis employing the single-molecule microscopy method TOCCSL revealed the disappearance of higher-order oligomers (such as trimers and tetramers) upon cholesterol depletion, which initially seemed contradictory to the FRET results. However, we reconciled this inconsistency using the FRAP method, which demonstrated a decreased mobile fraction of SERT in cholesterol-depleted cells. The combined data from FRET, TOCCSL, and FRAP experiments imply that cholesterol removal promotes the aggregation of trimers and tetramers into higher oligomeric structures, leading to increased FRET levels. These structures become immobilized, resulting in the decreased mobile fraction observed by FRAP microscopy. TOCCSL experiments specifically capture the mobile fraction with immobilized or slowed-down molecules not contributing to the analysis [72]. Unlike the immobile fraction, the mobile fraction of SERT appears to be more prevalent in its monomeric form when cholesterol levels are reduced.

In line with previous studies, we found substrate uptake through SERT, DAT, and NET, respectively reduced upon cholesterol depletion [18, 21, 28, 63]. By conducting confocal microscopy experiments, we could exclude a lower surface expression of these transporters as the cause of diminished substrate uptake. It has been shown that cholesterol binds to the conserved cholesterol site 1 in a hydrophobic groove between TM1a, TM5, and TM7 [17, 22]. Several studies suggested that cholesterol alterations affect the conformational equilibrium of the MATs, whereby depletion resulted in an increase in the fraction of transporters in the inward-facing conformation. Especially TM1a plays an important role in the conformational effects of cholesterol since this region undergoes large conformational changes in LeuT in the transition from outward-facing to inward-facing conformation [73, 74]. Cholesterol replenishment on the other hand increased the dwell time in the outward-facing conformation [18–20, 22, 23, 64]. In our study, cholesterol depletion resulted in a reduced binding affinity of orthosteric inhibitors at all tested MATs, which is in line with the MATs adopting the inward-facing conformation more frequently. However, cholesterol replenishment increased the inhibitor binding affinity to DAT and NET but not to SERT. This may be due to untreated SERT predominantly dwelling in the outward-facing conformation such that an increase in membrane cholesterol had no major influence on the conformational equilibrium. Electrophysiological recordings of currents mediated by SERT revealed that cholesterol depletion decreases the EC_50_ value for current induction. This observation aligns with a previous report that found a negative correlation between the potency of 5-HT at SERT and membrane cholesterol levels [22]. Our electrophysiological assessment (i.e., the two pulse protocols) indicated that the substrate turnover rate remained constant at reduced cholesterol levels. This finding seems to contradict the decreased V_max_ values observed in substrate uptake experiments. One plausible explanation for this inconsistency is that at lower cholesterol levels transporters are divided into two groups: one group that cannot find a cholesterol molecule for binding and remains stuck in the inward-facing conformation and another group that retains the ability to undergo full cycles with unchanged velocity because it can hold on to the remaining cholesterol. The existence of different SERT subpopulations due to cholesterol depletion has previously been described by Bjerregard et al. [64]. In electrophysiological recordings, only the second group is visible while the first is electrically silent. This contrasts with the other approach to determine the substrate turnover rate where the number of transporters needs to be counted through inhibitor binding experiments. In binding experiments, the high-affinity inhibitor will eventually bind all transporters including those that were originally inward-facing. As a result, information about the proportion of transport-incompetent transporters is lost.

To date, only one study has shown a reducing effect of cholesterol depletion on DAT-mediated efflux [23], which is in line with our data. A perplexing observation, however, is that reduced cholesterol levels affect substrate release through SERT differently than through DAT and NET. A possible explanation is that SERT can form higher-order oligomeric states than DAT and NET. While DAT and NET are mainly composed of dimers [31, 75], SERT can adopt trimers, tetramers, and higher-order oligomers [30, 41]. It has been shown that the N-terminus of the MATs is a lever required for the action of amphetamines [62]. It is tempting to speculate that, because of limited space, the N-terminus adopts a different shape in a higher-order oligomeric state compared to when it’s in a monomer or dimer form. Since the N-terminus plays a key role in amphetamine-induced substrate release [39, 40, 62], this could explain why substrate efflux increases in SERT when larger complexes form due to cholesterol depletion.

Based on our findings, we expect that changes in cholesterol levels influence neuronal function [12]. It has been suggested that cholesterol is crucial for the development and maintenance of neuronal function and plasticity [76]. Accordingly, cholesterol aberrations have been suggested as the unifying cause of synaptic degeneration [77]. Reduced cholesterol levels have been reported in patients with major depression, borderline personality disorder, dissociative disorder, and antisocial personality disorder [78–82] all of which have been linked to SERT [1, 48, 49]. Hence, we hypothesize that the interaction between cholesterol and the MATs is necessary for fine-tuning the transporter function. Some of the symptoms observed in psychiatric patients may arise from changes in the fraction of monoamine transporters bound to cholesterol. Moreover, pharmacological modulation of PIP_2_ binding to SERT could rescue the effects of reduced cholesterol levels. Consequently, further research on how altered cholesterol levels affect MAT function could lead to novel treatment options for these psychiatric disorders.

## Supplementary information

Supplement

## Data Availability

Data supporting the findings are available from the corresponding author upon reasonable request.

## Code availability

The MATLAB source code for single molecule microscopy analysis is available at: https://github.com/schuetzgroup/TOCCSL_analysis.
