# Supplementary material for: Cell membrane cholesterol affects serotonin transporter efflux due to altered transporter oligomerization
Source: Mol Psychiatry. 2025 Sep 2;31(2):963–75. doi: 10.1038/s41380-025-03201-y (PMC12815673; doi:10.1038/s41380-025-03201-y)
Supplement: Supplementary file 1 — Supplement [file 41380_2025_3201_MOESM1_ESM.pdf]

Supplement

Cell membrane cholesterol affects serotonin transporter efflux due to altered transporter oligomerization

Deborah Rudin, PhD,[1] Dino Luethi, PhD,[1,2] Marco Niello, PhD,[1,3] Jae-Won Yang, PhD,[1] Isabella Burger, MSc,[4] Walter Sandtner, PhD,[1] Ruth Birner-Gruenberger, PhD,[4,5] Gerhard J. Schütz, PhD,[2] Harald H. Sitte, MD,[1,6,7]*

[1]Institute of Pharmacology, Center for Physiology and Pharmacology, Medical University of Vienna, Waehringer Strasse 13A, 1090 Vienna, Austria

[2]Institute of Applied Physics, TU Wien, Lehargasse 6, 1060 Vienna, Austria

[3]Genetics of Cognition Laboratory, Neuroscience Area, Istituto Italiano di Tecnologia, Genova, Italy.

[4]Institute of Chemical Technologies and Analytics, TU Wien, Getreidemarkt 9, 1060, Vienna, Austria.

[5]Diagnostic and Research Institute of Pathology, Medical University of Graz, Neue Stiftingtalstrasse 6, 8010 Graz

[6]Hourani Center for Applied Scientific Research, Al-Ahliyya Amman University, Amman, Jordan

[7]Center for Addiction Research and Science-AddRess, Medical University Vienna, Waehringer Strasse 13A, 1090, Vienna, Austria

Running title: Cholesterol alters serotonin transporter efflux

***Correspondence**

Harald H Sitte, harald.sitte@meduniwien.ac.at

Medical University of Vienna, Center for Physiology and Pharmacology, Institute of Pharmacology, Waehringer Strasse 13a, A-1090 Vienna, Austria

Keywords: cholesterol, cell membrane, monoamine, transporter, oligomerization

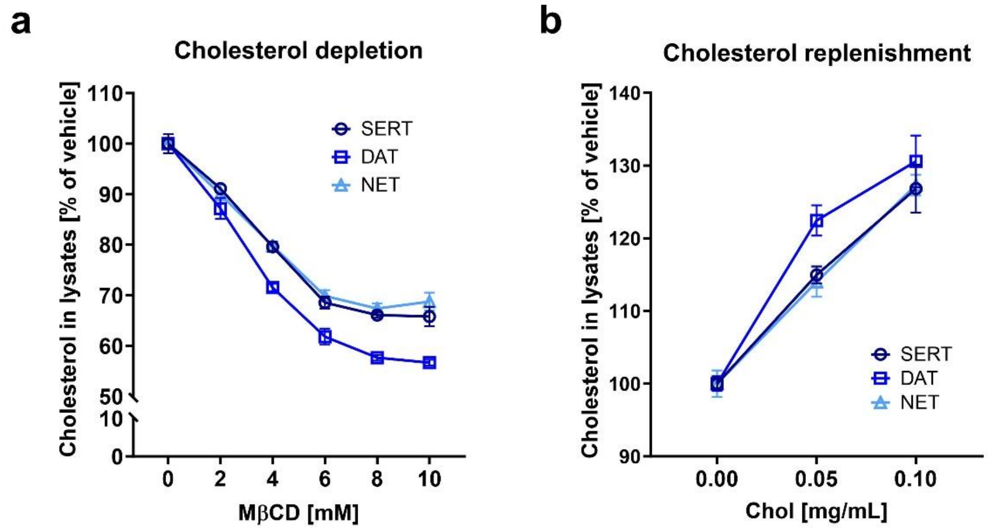

Suppl. Fig. S1. Quantification of cholesterol depletion and replenishment. Change in cholesterol content of HEK293 cells stably expressing SERT, DAT, and NET after pretreatment with MβCD for cholesterol depletion **a)**, or cholesterol (Chol) for cholesterol replenishment **b)** at the indicated concentrations for 30 min at 37 °C. Subsequently, the cellular cholesterol content was analyzed.

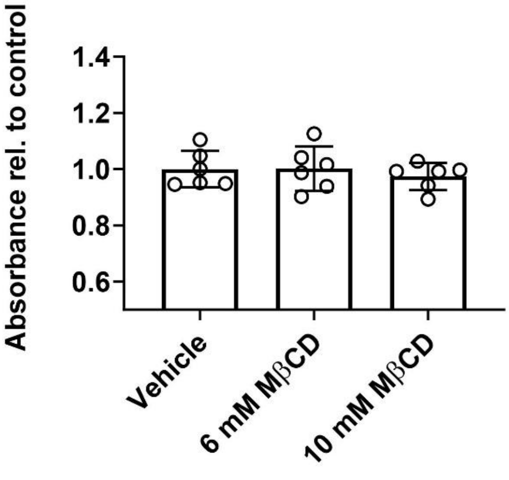

Suppl. Fig. S2. Cytotoxicity of cholesterol depletion. Potential cytotoxic effects of cholesterol depletion were assessed with the CellTiter 96® Non-Radioactive Cell Proliferation Assay (MTT) according to the manufacturer's protocol. The cells were incubated with 6 and 10 mM MβCD or vehicle control for 30 min at 37 °C. Data are expressed as mean ± SD.

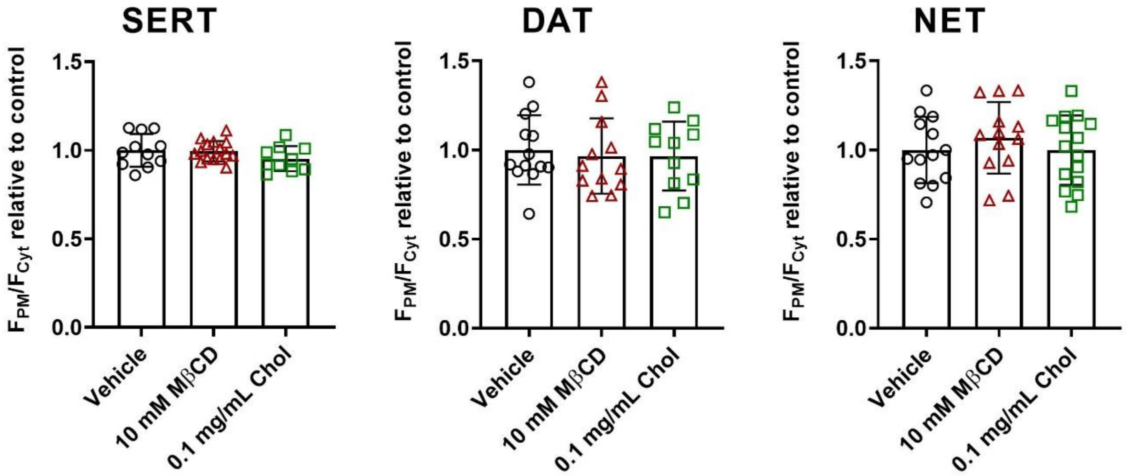

Suppl. Fig. S3. Monoamine transporter internalization after cholesterol alterations. Plasma membrane fluorescence (F_{PM}) relative to cytosolic fluorescence (F_{Cyt}) of YFP-tagged SERT, DAT, and NET after treatment with 10 mM MβCD, 0.1 mg/mL Chol, or vehicle control for 30 min. Data are expressed as mean ± SD of 10–15 cells per condition.

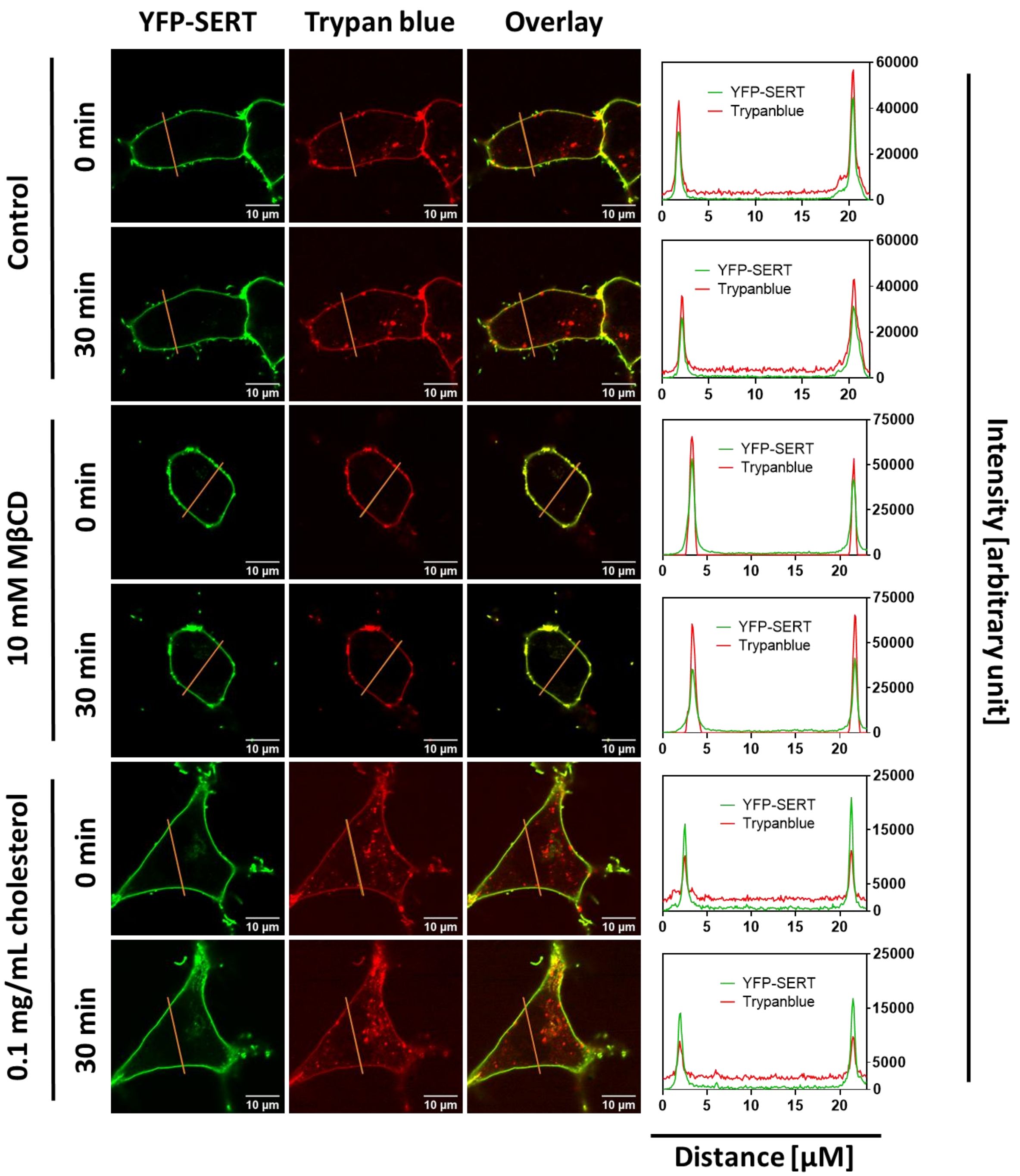

Suppl. Fig. S4. Cell membrane expression of SERT. Confocal images of YFP-SERT stained with 0.4% trypan blue. The orange line indicates where intensity relief depicted on the right was recorded.

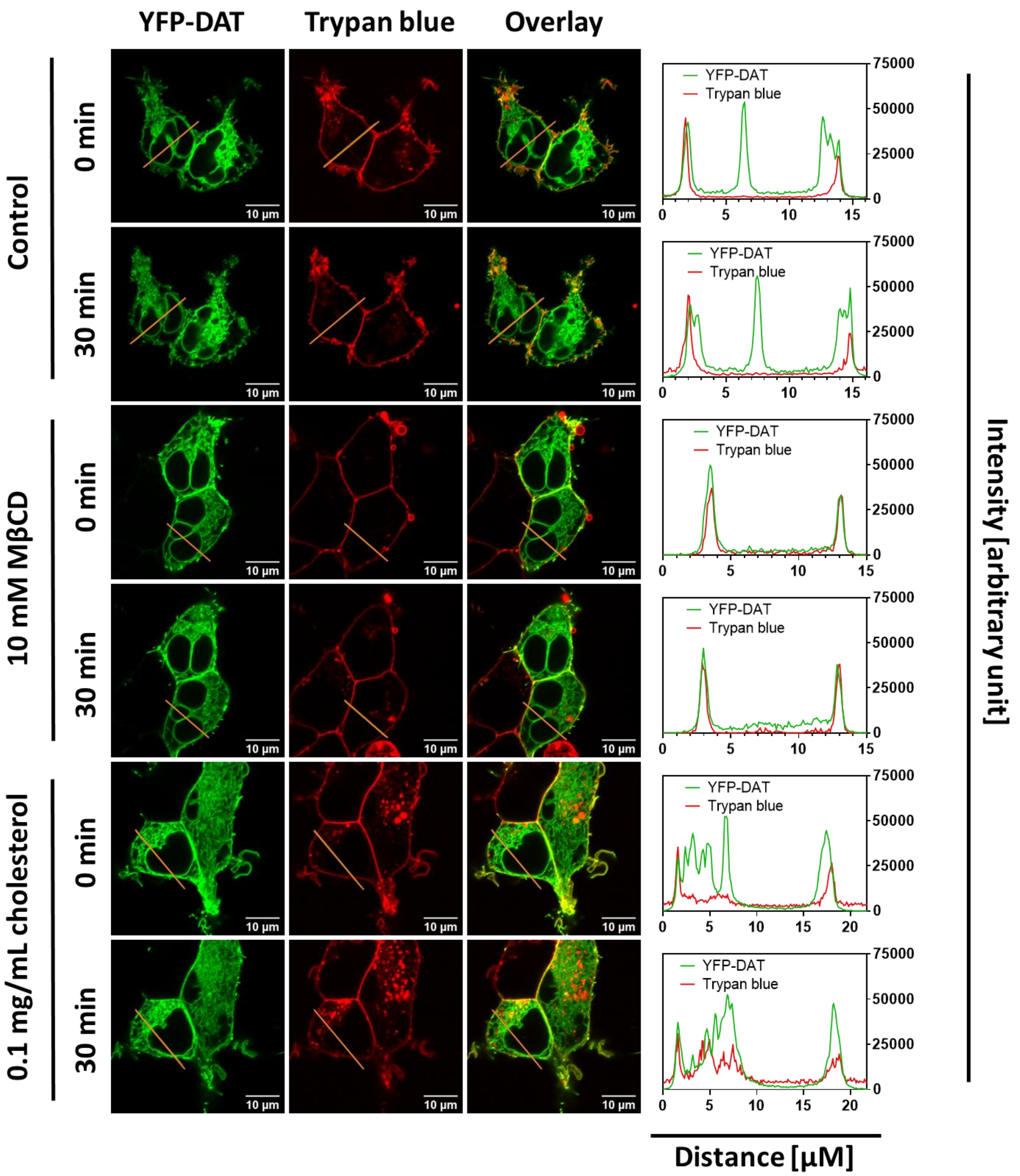

Suppl. Fig. S5. Cell membrane expression of DAT. Confocal images of YFP-DAT stained with 0.4% trypan blue. The orange line indicates where intensity relief depicted on the right was recorded.

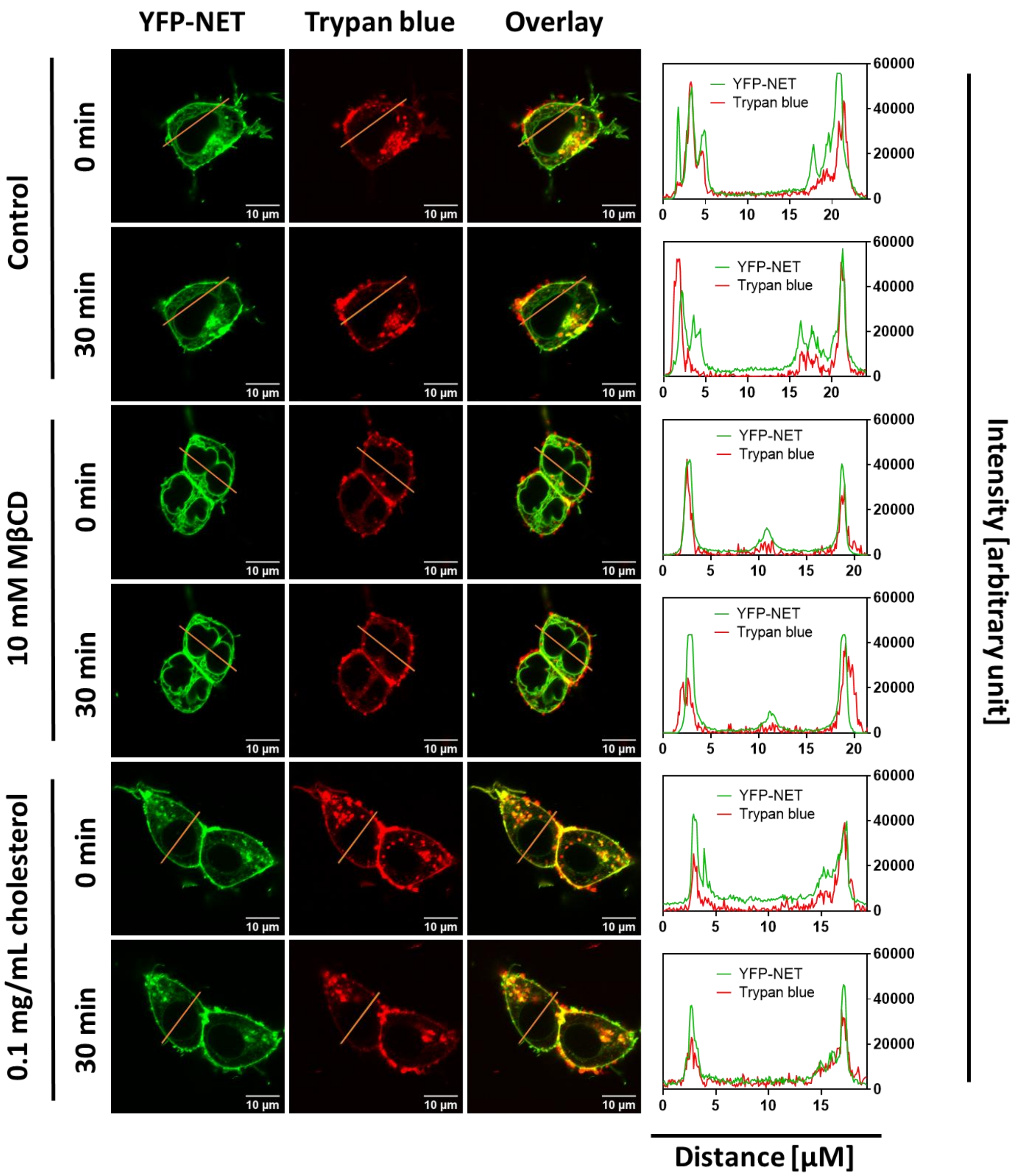

Suppl. Fig. S6. Cell membrane expression of NET. Confocal images of YFP-NET stained with 0.4% trypan blue. The orange line indicates where intensity relief depicted on the right was recorded.

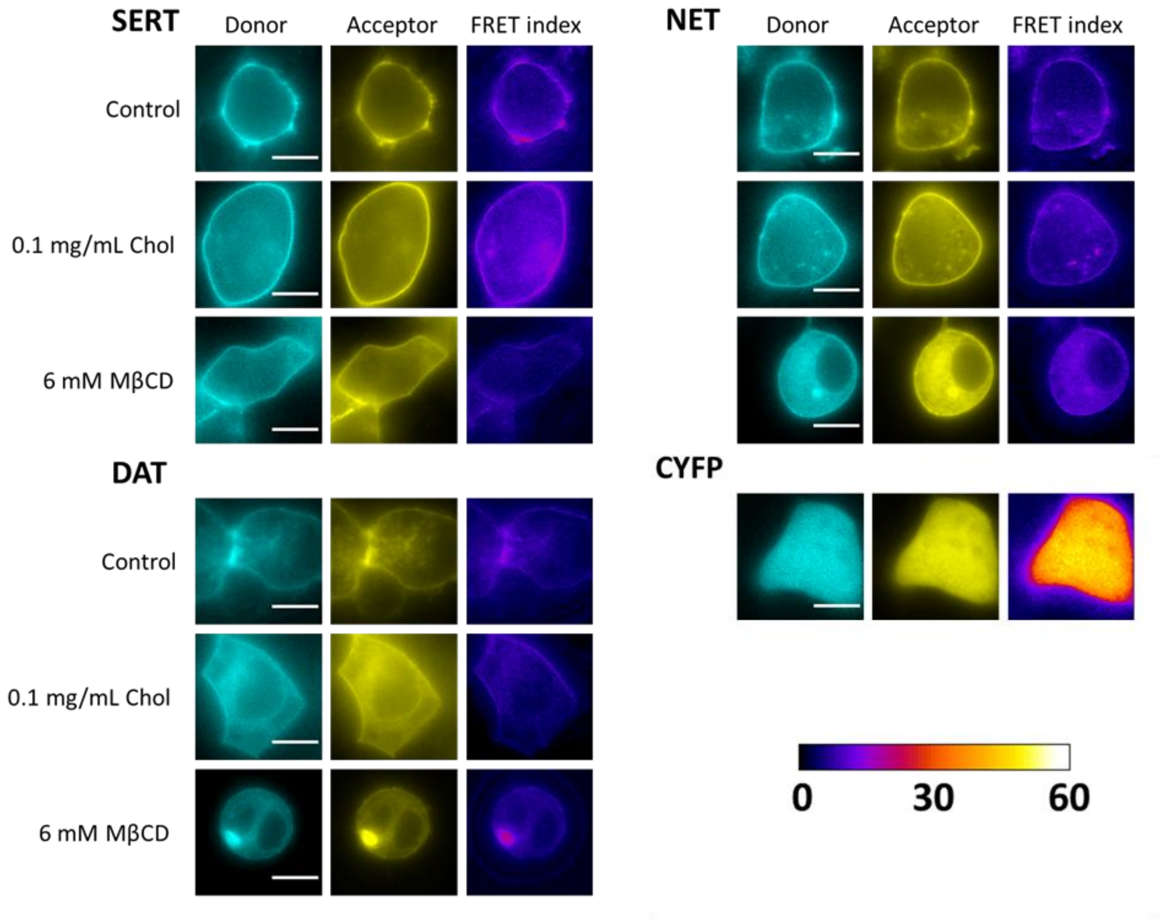

Suppl. Fig. S7. Representative CFP (donor), YFP (acceptor), and FRET images for each transfected cell line and condition. The FRET index was computed after background subtraction. The scale bar measures 10 µm.

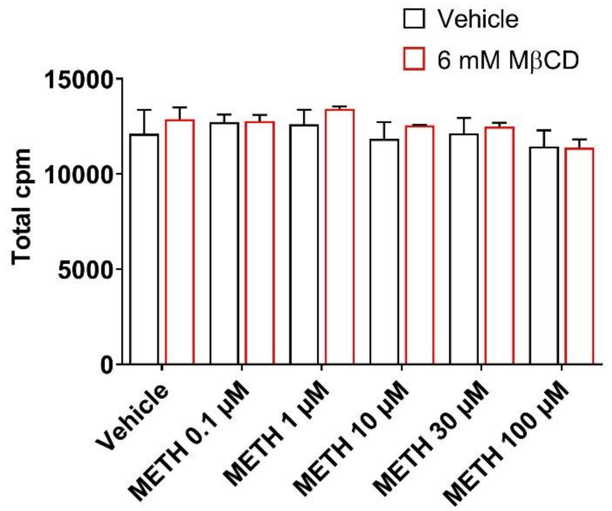

Suppl. Fig. S8. Effect of MβCD on total radioactivity (counts per minute, cpm) measured at the end of the SERT release experiment. Substrate release in HEK293 cells expressing SERT was induced by methamphetamine (METH) in the absence (white) or presence (red) of 6 mM MβCD. The bar graph shows the mean ± SD of one representative experiment performed in triplicate.

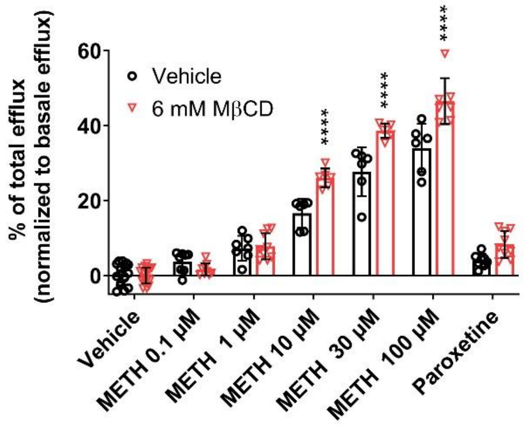

Suppl. Fig. S9. Effect of cholesterol on YhSERT efflux with different substrate MPP⁺. Transporter-mediated efflux in HEK293 cells expressing SERT. Substrate release was induced by *d*-methamphetamine (METH) in the absence (black bars) or presence (red bars) of 6 mM MβCD after preloading the transporter-transfected cells with radiolabeled MPP⁺. The transporter blocker paroxetine was used to assess non-specific efflux. Data are expressed as mean ± SD from at least three experiments performed in triplicate. Data were analyzed using two-way ANOVA followed by Sidak's multiple comparison test. *, **, and *** indicate $P < 0.05$, $P < 0.01$, and $P < 0.001$, respectively, when compared to the corresponding condition without MβCD.

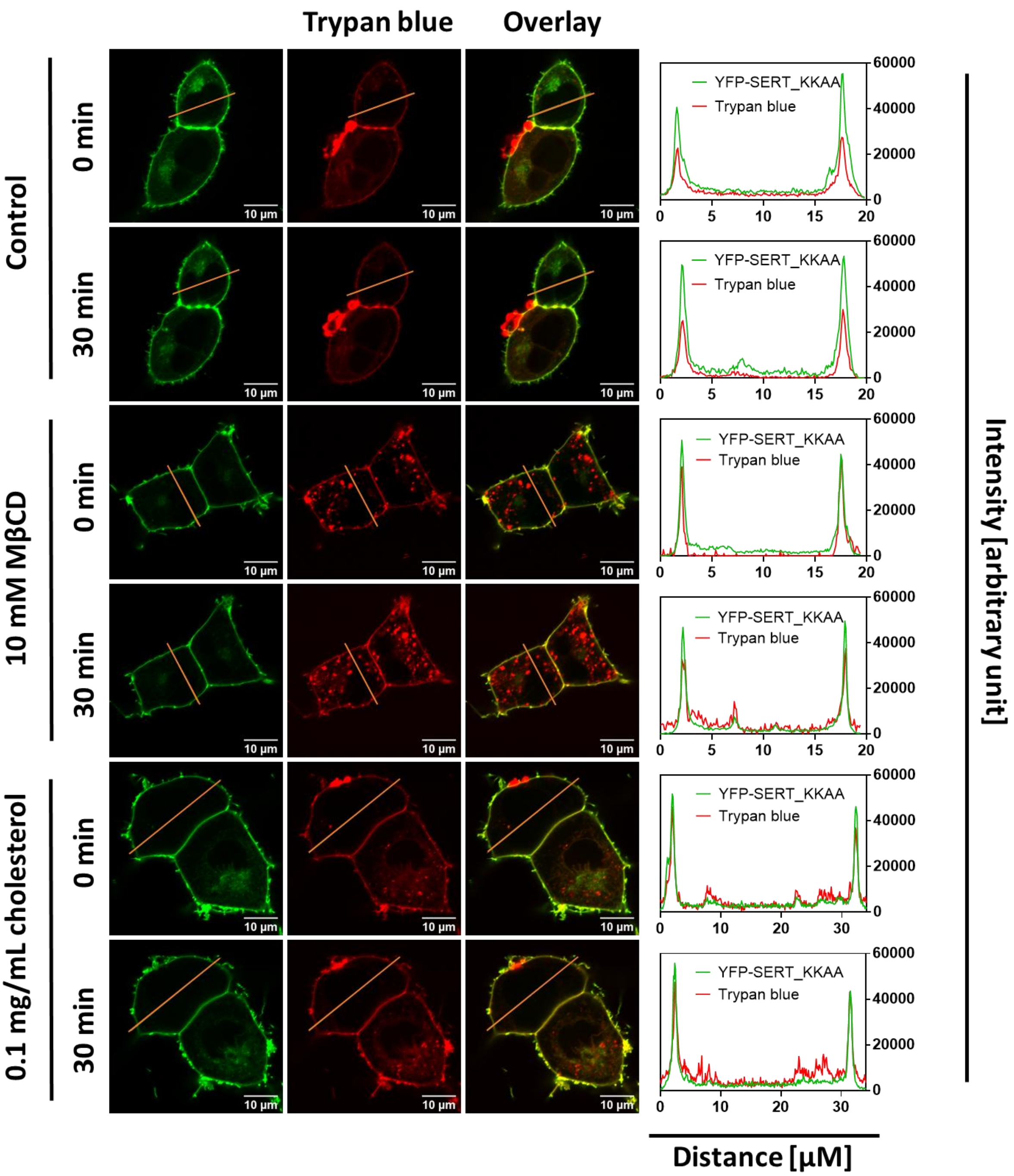

Suppl. Fig. S10. Cell membrane expression of SERT_KKAA. Confocal images of YFP-SERT_KKAA stained with 0.4% trypan blue. The orange line indicates where intensity relief depicted on the right was recorded.
